# Theory of Mind and Parental Mental-State Talk in Children with CIs

**DOI:** 10.1093/deafed/enad004

**Published:** 2023-03-23

**Authors:** Agnieszka Pluta, Magdalena Krysztofiak, Małgorzata Zgoda, Joanna Wysocka, Karolina Golec, Katarzyna Gajos, Tadeusz Dołyk, Tomasz Wolak, Maciej Haman

**Affiliations:** Faculty of Psychology, University of Warsaw, Warsaw, Poland; Bioimaging Research Center, World Hearing Center, Institute of Physiology and Pathology of Hearing, Warsaw, Poland; Faculty of Psychology, University of Warsaw, Warsaw, Poland; World Hearing Center, Institute of Physiology and Pathology of Hearing, Warsaw, Poland; Faculty of Psychology, University of Warsaw, Warsaw, Poland; Faculty of Psychology, University of Warsaw, Warsaw, Poland; Faculty of Psychology, University of Warsaw, Warsaw, Poland; Faculty of Psychology, University of Warsaw, Warsaw, Poland; Bioimaging Research Center, World Hearing Center, Institute of Physiology and Pathology of Hearing, Warsaw, Poland; Faculty of Psychology, University of Warsaw, Warsaw, Poland

## Abstract

Previous studies have suggested that parents may support the development of theory of mind (ToM) in their child by talking about mental states (mental state talk; MST). However, MST has not been sufficiently explored in deaf children with cochlear implants (CIs). This study investigated ToM and availability of parental MST in deaf children with CIs (*n* = 39, *M_age_* = 62.92, SD = 15.23) in comparison with their peers with typical hearing (TH; *n* = 52, *M_age_* = 52.48, SD = 1.07). MST was measured during shared storybook reading. Parents’ narratives were coded for cognitive, emotional, literal, and non-mental references. ToM was measured with a parental questionnaire. Children with CIs had lower ToM scores than their peers with TH, and their parents used more literal references during shared storybook reading. There were no significant differences in the frequencies of cognitive and emotional references between groups. Parental emotional references contributed positively to children’s ToM scores when controlling for the child’s age and receptive grammar only in the CI group. These results indicated some distinctive features in parents of deaf children with CIs’ MST and highlighted the role of MST in the development of ToM abilities in this group.

Theory of Mind (ToM) is defined as an ability to ascribe mental states (such as desires, emotions, beliefs) to oneself and others to predict and explain behavior based on these states (Premack & Woodruff, 1978). Children typically advance in this capacity until later childhood. However, the substantial shift in ToM development occurs in children between the ages of 3 and 5 years, when they begin to explicitly manifest their understanding that the mental states of others may differ from their own and from reality (Wellman, 2018; Wysocka et al., 2020). The false belief task is regarded as a litmus test of this ability (Dennett, 1978).

The child’s language abilities (Farrar, Benigno, Tompkins, & Gage, 2017) and certain aspects of their family environment, particularly their family structure (e.g., number of siblings, the family’s socio-economic status) and parental mental state talk (MST), can influence ToM development, accounting for inter-individual differences in typically developing children (Devine & Hughes, 2018; Tompkins, Benigno, Kiger Lee, & Wright, 2018; Tompkins, Montgomery, & Blosser, 2021).

MST may be operationalized as the quantity and quality of references to mental states that parents make during their everyday interactions with children. The relationship between MST and a child’s ToM development may be explained using social constructivist accounts. These propose that parents support their child’s social understanding semantically (through exposure to mental state vocabulary; e.g., “think,” “claim,” “knows”), syntactically (through exposure to complement structures; e.g., “John thinks that Marry knows something”) and pragmatically (through exposure to someone else's communicative intentions) (Carpendale & Lewis, 2004; Nelson et al., 2003).

Parental MST advances along with the child's development: parents talk first about desires and needs, then about emotions and finally about unobserved cognitive mental states such as intentions or beliefs (Bartsch & Wellman, 1995; Bretherton & Beeghly, 1982; Brown & Dunn, 1991; Jakubowska, Szpak, & Białecka-Pikul, 2018; Taumoepeau & Ruffman, 2008). This pattern seems to reflect the developmental trajectory of ToM, as children first acquire the ability to attribute desires and basic emotions to another person, and only later do they develop an explicit understanding of their own and others’ minds (Wellman, 2018).

Previous studies have demonstrated a positive link between MST and ToM in typically developing children (for review and metaanalysis, see: Tompkins et al., 2018). This phenomenon is robust and seems to be generalizable to both Western and non-Western samples (Chan, Wang, Devine, & Hughes, 2020). For example, Devine and Hughes (2018) found a significant modest association between MST and ToM mastery (measured by children's scores on a battery of false belief tasks). The same researchers have also conducted a longitudinal study (Devine & Hughes, 2019) with over one hundred preschoolers and their parents, finding that parental MST (measured as the frequency of references to cognitions, emotions, and desires during shared storybook reading and toy play) predicted the child’s false belief understanding one year later. This result was significant even after controlling for the child’s age, parental verbosity (i.e., total number of words), and education. Slaughter, Peterson, and Mackintosh (2007) investigated both the quantity and quality of mothers’ MST during shared storybook reading and its relationship with the child’s (aged 3 and 4 years) ToM performance. They found that maternal clarifying talk that explains unobservable cognitive mental states (e.g., characters’ thoughts) was positively related to the child’s false belief understanding.

In summary, previous studies indicate a positive relationship between parental MST and the ToM abilities in children with typical development and typical hearing levels. The general idea behind this association is that specific types of words and/or utterances draw attention to mental concepts and accentuate as well as contextualize the psychological perspectives of different people. In this context, it is important to investigate the relationship between MST and ToM in children who are at risk of language development delays and might have less than optimal access to rich conversations including MST. Thus, examining this relationship in a population of deaf children who are raised by hearing parents communicating in spoken language (Deaf of Hearing; DoH) is of special significance.

Taking into account that language acquisition (spoken or signed) and access to mind-related conversational input are deeply intertwined with ToM development, it is not surprising that DoH children are reported to experience problems with false belief understanding (Ketelaar, Rieffe, Wiefferink, & Frijns, 2012; Peterson, 2020; Schick, de Villiers, de Villiers, & Hoffmeister, 2007; Sundqvist, Lyxell, Jönsson, & Heimann, 2014; Yu, Stanzione, Wellman, & Lederberg, 2021). The results of studies on this subject are largely consistent, regardless of the research methods used—traditional measures such as false belief tasks (Walker, Ambrose, Oleson, & Moeller, 2017) or those modified to minimize verbal demands (Pluta et al., 2021).

It is only recently that researchers addressing this issue have begun to recruit DoH who received cochlear implants (CIs) under 12 months of age (Holcomb & Smeal, 2020). Benefits of early cochlear implantation towards more typical auditory development are presently unquestionable (for a review see Sharma, Cushing, Papsin, & Gordon, 2020). They are, however, not uniform in the area of expressive and receptive language. Some children attain a level of oral language development equal to that of their peers with typical hearing by the time of school entry while others continue to experience delays in this respect (Boons et al., 2013; Geers, Moog, Biedenstein, Brenner, & Hayes, 2009; Lorens, Obrycka, & Skarżyński, 2020; Paluch et al., 2019; Peterson, 2004; Zgoda, Lorens, & Skarzynski, 2012).

The handful of studies on ToM development in children who received CIs early indicate that implantation has also a positive effect on ToM abilities in DoH (Sundqvist et al., 2014). Alas, it does not imply a typical temporal trajectory of ToM development for most deaf children with CIs. For example, our previous study (Pluta et al., 2021) showed that explicit understanding of false beliefs was delayed by about 1.5 years in most children with CIs, meaning that they mastered the false belief task by age 6, rather than, like most hearing peers, between 4 and 5 years of age.

Although researchers are acknowledging the role of mind-oriented conversations in the development of ToM (Marschark, Edwards, Peterson, Crowe, & Walton, 2019), there are a few studies that have investigated parental MST directed towards deaf or hard of hearing children (Dirks, Stevens, Kok, Frijns, & Rieffe, 2020; Moeller & Schick, 2006; Morgan et al., 2014) and to the best of our knowledge, only one that examined the relationship between MST and ToM directly in deaf preschoolers with CIs (Moeller & Schick, 2006).

These few studies reported that parents of children with hearing loss used less mental state references (especially references to cognitions) than those of children with typical hearing (Dirks et al., 2020; Moeller & Schick, 2006; Morgan et al., 2014). Moeller and Schick (2006) found that mothers’ MST predicted deaf (but not hearing) children’s false belief understanding, even after controlling for the child’s age and language abilities. However, these results cannot be easily generalized to DoH, who use only spoken language and receive hearing aids at an early age, as the study group was strongly heterogeneous in terms of hearing aids and the age at which the children started using them (10 used CIs, 10 hearing aids, 2 did not use hearing aids; CI implantation occurred between 36 and 96 months of age) and mode of communication (sign or spoken language).

Interestingly, while Morgan et al. (2014) found that mothers of deaf infants (aged 12–19 months) used fewer cognitive mental state terms (such as “think” or “know”) during conversational turn-taking than mothers of infants with typical hearing, there were no differences between the groups in terms of the frequency of desire and emotion references. According to the authors, as understanding of desires and emotions precedes understanding of the concept of belief (Peterson & Wellman, 2019), by using simplified conversations that pertain to observable states (e.g., facial expressions) the mothers could have matched their mind-oriented talk to the anticipated capacities of their children. This implies that caregivers begin discussing mental states with their children when they demonstrate an understanding of these concepts. This capacity, as the previous studies indicated, occurs later in DoH than in the case of hearing peers (Moeller & Schick, 2006; Morgan et al., 2014). Hence, it is also plausible that hearing caregivers of deaf children begin making references to unobservable states later than parents of hearing children.

The relationship between MST and ToM development can, of course, be mediated by the child's expressive language abilities. This means that the child benefits from MST in accordance with his or her language abilities. Therefore, parents of children with better expressive language capacities tend to use more words and longer utterances. In line with this, Dirks et al. (2020) demonstrated a positive relationship between quantity and quality measures of parents’ linguistic input (more words, longer utterances, and more mental state language) and the expressive language abilities of toddlers with moderate hearing loss.

In conclusion, despite the apparent interest of researchers in the relationship between MST and ToM in deaf children who are CI users, this topic is not well explored in this population. In particular, this relationship has not been studied whatsoever in children, who use cochlear implants (CIs) from an early age and they are raised by parents communicating only in spoken language. This is surprising given that these studies are crucial for elucidating the mechanisms underlying delayed acquisition of ToM-related abilities in children with CIs. In addition, if an association between MST and ToM is demonstrated, this may be useful for developing MST-based therapeutic programs aimed at supporting ToM development. Moreover, the conclusions of previous studies are limited by the fact that they used samples heterogeneous in terms of hearing prosthesis (CIs or hearing aids), the families’ communication modality options (ranging from exclusively auditory, through bilingual/bicultural, to exclusively visual), and onset of CI or hearing aid use. Furthermore, ToM has only been examined in clinical settings. Thus, little is known about how deaf children with CIs employ knowledge of the mental states of others in everyday situations.

Therefore, in an attempt to fill this gap in the literature, the present study had the following aims: a) to compare a wide range of ToM abilities in children with CIs and their peers with typical hearing (TH) using a caregiver-informant measure that gauges how a child performs in everyday situations that require attribution of mental states to others; b) to compare the frequency of parental references to mental states between parents of deaf children with CIs and parents of children with TH; c) to investigate the relationships between parental MST and ToM in a sizeable sample of DoH with CIs and their TH peers; and finally d) to examine the contribution of MST to ToM abilities in children with CIs.

In contrast to previous research, the studied group was more homogenous: all deaf children were CI users who communicated only in spoken language and neither parents nor children were familiar with sign language. Moreover, all children received their first CI between 5 and 20 months of age. Additionally, in order to investigate ToM development, a new caregiver-informant measure (the Theory of Mind Inventory) designed to tap into a wider range of the children's ToM competencies than just false belief understanding was applied. This measure was recently shown to be a valid tool for identifying ToM challenges in children with hearing loss and its scores correlate with scores for receptive vocabulary and pragmatic language development (Hutchins, Allen, & Schefer, 2017; Hutchins, Prelock, & Bonazinga, 2012). In addition, given that classical ToM tests have been criticized for their excessive complexity and receptive language requirements (Bloom & German, 2000), we recognized the great importance of using a measure that is relatively free of these limitations.

Also, in contrast to some other studies that analyzed frequencies of mental state verbs (i.e., the number of words such as “think,” “know,” “feel,” etc.; e.g., Morgan et al., 2014), all utterances were evaluated. This was driven by previous research findings showing that explanatory talk about mental states (e.g., “He acted in a certain way because he did not know something”) may be particularly closely related to children's ToM understanding (Ruffman, Slade, & Crowe, 2002; Slaughter et al., 2007).

Finally, apart from the previously proposed categories of coding MST (i.e., references to cognitions, emotions, desires, or perceptions), we additionally looked at non-mental references rather than analyzing them together as an alternative to MST (Moeller & Schick, 2006).

In line with the most recent research findings reviewed above, the following hypotheses were proposed:

1) Children with CIs will have lower performance than their peers with typical hearing in everyday situations that require understanding another person's psychological perspective.
2) During conversations with their children, parents of deaf children with CIs will use fewer inferential utterances referring to mental states (cognitions and emotions).
3) There will be a positive relationship between parental MST (references to emotions and cognitions) and ToM abilities in both groups.
4) Parental MST will contribute to the child’s ToM skills, when controlling for the child’s language skills and age.

## Methods

### Participants

The study sample consisted of 100 parent–child (mother or father) dyads divided into two groups: children with CIs and their parents and children with TH and their parents. Parents of deaf children with CIs were contacted through the clinics by an educator of the deaf and hard of hearing. Children with TH were recruited via advertisements posted on social media. All parents had typical hearing and used Polish to communicate with their children. None of the parents used Polish Sign Language. Based on parental responses to survey questions, none of the children with TH or CIs had any diagnosis of developmental, systemic, genetic, or metabolic disorders, or major medical complications during pregnancy or delivery. Written informed consent was obtained from the parents/caregivers of the children participating in the study. All children agreed to take part in the study. Recruitment and experimental protocols were approved by the Ethics Committee of the University of Warsaw and were conducted in accordance with the World Medical Association’s Declaration of Helsinki.

#### Deaf children with cochlear implants (CI Group)

Forty-three children with CIs and their parents participated in the study. Data from four children were excluded either because parents only read the book and did not provide any extratextual utterances or the child was not interested in shared reading (*M_age_* = 51.25, SD = 13.15). The final sample consisted of 39 children with CIs and their parents, including 29 mothers and 10 fathers. The children’s ages ranged from 35 to 94 months (*M_age_* = 62.92, SD = 15.23). All children had a history of congenital bilateral profound sensorineural and prelingual hearing loss (hearing loss over 90 dB in the better ear). The children were raised in a hearing culture and did not use sign language to communicate. All children received their first CI between 5 and 20 months of age. Seventy two percent of the children were reported to wear their CI(s) regularly with an average time of around 12 hours per day (*M* = 12.09, SD = 1.74). For other children, parents reported CI(s) use of minimum 10 hours per day. Descriptive statistics related to CIs are presented in Table 1.

**Table 1:** Descriptive statistics related to children with CIs

	*n*	Range	*M*	SD
Age at first CI (months)	39	5–20	11.18	3.64
Age at second CI (months)	31	10–67	39.74	15.04
First CI duration (months)	39	23–85	51.38	16.35
Second CI duration (months)	31	3–67	24.77	19.14

*Note*. CI = Cochlear implant

#### Children with typical hearing (TH group)

Fifty-seven children with typical hearing and their parents participated in the study. Data from five children were excluded either because parents only read the book and didn’t provide any extratextual utterances or the child was not interested in shared reading (*M_age_* = 47.60, SD = 9.96). The final sample consisted of 52 children with typical hearing levels and their parents, including 42 mothers and 10 fathers. The children's ages ranged from 36 to 70 months (*M_age_* = 52.48, SD = 1.07).

Descriptive statistics for both groups are presented in Table 3.

### Procedures

This study was a part of a larger project. Examinations were conducted by four qualified psychologists experienced in working with children, including those with CIs. These were carried out individually in quiet clinical (CI group) and laboratory settings (CI and TH groups) at the Institute of Physiology and Pathology of Hearing or in the laboratory at the Faculty of Psychology at the University of Warsaw. For some of the children, both from the CI and TH group, the testing was split into two 45 to 60 minute sessions (spread approximately over 2 weeks, *M_days_* = 15.83, SD = 29.28).

First, children were given the sentence comprehension subtest from the Test of Language Development (Test Rozwoju Językowego, TRJ; Smoczyńska et al., 2015). Then, children’s general intelligence was tested with the use of the Abbreviated Battery of Stanford-Binet Intelligence Scales, fifth edition (SB5; Roid, Sajewicz-Radtke, Radtke, & Lipowska, 2017). While the children participated in testing, parents filled in a questionnaire on the child's ToM abilities (Theory of Mind Inventory-2; Hutchins & Prelock, 2016) and a questionnaire concerning the parents’ education and child’s health. Finally, parents were asked to read a storybook with their child. The children received a small gift (a book or a toy) for their participation in the study.

#### Receptive grammar—Sentence Comprehension

Children’s receptive grammar was assessed with the Sentence Comprehension subtest from the TRJ (Smoczyńska et al., 2015)—a standardized and normed measure of language skills for children aged from 4;00 to 8;11 years. The Sentence Comprehension subtest was based on the Test for Reception of Grammar (TROG; Bishop, 2003). It included 32 cards with 4 different pictures on each board. Participants were presented with 32 syntactically complex sentences, which they were asked to match to the corresponding picture from the set of four pictures (e.g., the hen is watching the duck, which is flying). Participants can obtain 32 points in total, one for each sentence. The reliability of this subtest, assessed with Cronbach’s α, is high (.80-.91, depending on the age group). Validity of the TRJ was demonstrated with several measures, including the measures of phonological memory and processing and fluid intelligence. Norms for TRJ were prepared on a representative sample of 1,800 children in Poland (Smoczyńska et al., 2015).

#### General intelligence

General intelligence was assessed with the Polish adaptation of the Abbreviated Battery of Stanford-Binet Intelligence Scales, fifth edition (SB5; Roid et al., 2017) which is based on two subtests: verbal (knowledge, verbal brief IQ) and nonverbal (fluid reasoning, nonverbal brief IQ). SB5 was included to control for the participants’ general cognitive abilities. The reliability of the brief IQ measure of SB5, measured with split-half reliability using the Spearman-Brown formula, is high (.92), for children aged from 4 to 8 years. Validity of SB5 in children from 4 to 8 years has been demonstrated with correlations with other measures of intelligence and language skills. Norms for SB5 were prepared on a representative sample of 2,350 participants aged from 2 to 19 years in Poland (Roid et al., 2017).

#### The Theory of Mind Inventory-2

The Theory of Mind Inventory-2 (ToMI-2; Hutchins & Prelock, 2016) was used to assess the children's ToM abilities. The ToMI-2 is a parental questionnaire and consists of 60 items divided into three subscales corresponding to the stages of ToM development (early, basic, and advanced). Together all three subscales constitute a combined ToM scale. The early ToM subscale measures the competences that are considered to be prerequisites of mental state understanding, such as shared attention and affect recognition (e.g., “My child recognizes when others are happy”). The basic ToM subscale captures the social understanding characteristic of typically developing preschool children, such as false belief understanding or mental state terms (e.g., “My child understands the word ‘think’,” “If I showed my child a cereal box filled with cookies and asked ‘What would someone who has not looked inside think is in the box?’, my child would say that another person would think that there was cereal in the box”). The advanced ToM subscale identifies complex social competences that appear in typical development between the ages of 6 and 8, such as second order understanding of belief, recursive thinking, and metapragmatic and metalinguistic skills (e.g., “My child understands that people often have thoughts about other peoples’ thoughts”).

The items in the questionnaires describe children's possible daily behaviors (“My child speaks differently to young children versus adults e.g., uses simple language or higher pitch when speaking to youngsters”) or their reactions to other people's behaviors (“If I looked up and stared at the sky, my child would also look up to see what I was looking at”) and do not require knowledge of the developmental stages of theory of mind.

The reliability of the ToMI-2, as assessed by Cronbach's α, is high (.98). The construct validity was determined by calculating the correlation between caregivers’ (*n* = 124) ratings on ToMI-2 and their children's (aged 2.0–12.8 years) performance on the ToM Task Battery (Hutchins, Prelock, & Chace, 2008). The analysis yielded a relatively high correlation (*r* = .66) indicating that parents reliably assessed their children's competence in inferring someone’s else's emotional and cognitive mental states (Hutchins & Prelock, 2016).

Parents were instructed to assess how well each statement describes their child (e.g., “My child understands whether someone hurts another on purpose or by accident”) by placing a mark at the appropriate point along a 0–20 point scale divided into the following options: *definitely not*, *probably not*, *undecided*, *probably*, and *definitely*. A Polish translation of the ToMI-2 scale was used in the study. All ToMI-2 scores were scaled to account for the different numbers of items on each scale.

#### Shared storybook reading

Similar to the study of Tompkins (2015), the parents (mother or father) were asked to read a book with their child: “Mr. Peek and the Misunderstanding at the Zoo” (in its Polish translation) by Kevin Waldron (2010). Parents had been instructed to read the book in the same way that they would read at home and they were not primed to focus on any particular aspect of it. All interactions were audiotaped for later transcription.

The choice of the book was motivated by its content, which provided many opportunities to discuss the mental states of the characters in the story. This book has also been used in other studies, including ToM research (Tompkins, 2015; Tompkins, Bengochea, Nicol, & Justice, 2017). It has also been translated into Polish (Waldron, 2013). In the story, children are introduced to Mr. Peek, the zookeeper, who, by mistake, puts on a jacket that is too small for him. He then does his rounds at the zoo. As he passes the animal enclosures, he talks to himself and makes negative comments (e.g., you have gotten very fat, you need to eat less), but the animals think he is talking about them. Finally, Mr. Peek realizes that he has switched jackets with his son and goes around the zoo again, this time making positive comments about himself. Understanding the plot of the book and its humorous elements requires that the child realizes that the animals do not know that Mr. Peek is talking to himself and that he does not know that he has swapped his jacket with his son.

#### Transcription and coding

Recordings were transcribed verbatim according to the Codes for the Human Analysis of Transcripts (CHAT) format and were then analyzed using Computer Language Analysis (CLAN) software (MacWhinney, 2000). This was done for calculating parents’ verbosity and to perform some additional analysis not presented in this study.

Coding was performed according to the scheme proposed by Tompkins et al. (2017) because: a) it provided a comprehensive representation of various topics that can occur in conversations with young children, and b) it has been previously used to analyze parent–child conversations in the context of the same book as used in our study. However, modifications were made to the coding scheme to tailor the analyses to the overarching goal of the study (i.e., we paid attention to expressions for mental, emotional, and nonmental states separately).

**Figure 1:**
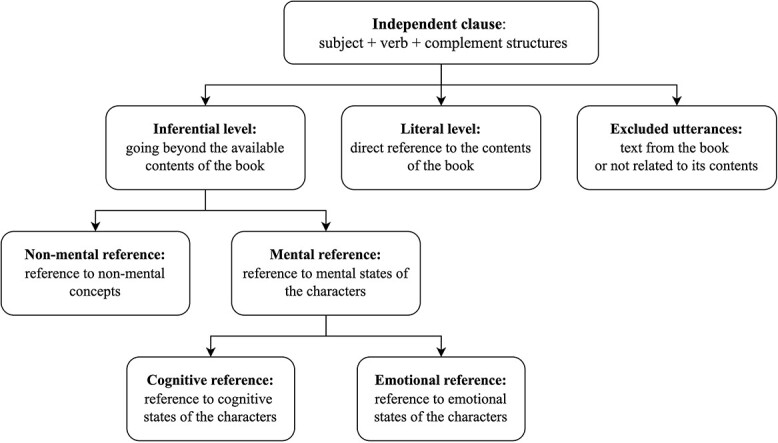
The coding scheme.

Firstly, following the guidelines of Tompkins et al. (2017), utterances were divided into independent clauses (i.e., subject + verb + complement structures). Only extratextual utterances relating to the story were coded. Utterances containing only the text directly read from the book or not related to the story (e.g., “will you sit and listen?”) were excluded, as in other studies (Tompkins, 2015; Tompkins et al., 2017).

Secondly, every clause was assigned to one of two levels of abstraction, following the coding scheme from Tompkins et al. (2017): 1) literal—referring directly to information available in the book (talk focused on the here and now) or 2) inferential—talk that goes beyond the here and now and which is more conceptual than perceptual.

Literal utterances included labeling, describing physical properties of the objects seen in the book, and counting (e.g., “this jacket is green,” “there are 3 penguins here”). Inferential utterances could include talk relating to previous experiences, predicting what will happen next in the story, referring to what occurred earlier in the story, drawing conclusions, hypothesizing, and giving definitions (e.g., “the hippos think that Mr. Peek is talking to them,” “Penguins are birds that cannot fly”).

Then, each inferential clause was assigned to the categories of *inferential mental* (when they referred to non-observable mental states) or *inferential non-mental* (when they did not refer to beliefs, desires, perspectives, etc.; e.g., “whales are mammals”).

Finally, inferential mentalistic utterances were further divided into *emotional* (referring to emotional states) and *cognitive* (referring to beliefs, state of knowledge, perspectives).

The coding scheme is shown in Figure 1. Examples of utterances assigned to each category can be found in Table 2.

**Table 2:** Coding categories and examples of parent’s utterances (translations from Polish)

Utterance category		Description	Example
Non-inferential	Literal utterances	Utterances with a general or selective description of an object, scene, or character.	“Look what the bear is doing.” “What is this face that the bear is making?”
Inferential	Emotional utterances	Utterances referring to the emotional states of the characters.	“It made her happy.” “He’s in a good mood now.”
	Cognitive utterances	Utterances referring to the cognitive states of the characters or emphasizing their cognitive perspective.	“But the bear thought it was about him.” “He was talking to himself all the time, wasn’t he?”
	Non-mental utterances	Utterances summarizing the course of the story, referring to the experience of the child or providing factual information.	“It’s because they changed their jackets!” “What do penguins usually eat?”

*Note.* The coding scheme was based on the model described by Tompkins et al. (2017), where a more precise description of the literal and inferential levels can be found.

Transcriptions were coded independently by three judges. Agreement was calculated for 15% of the transcripts. There was good to excellent agreement between the coders calculated with the intra-class correlation (ICC; two-way mixed effects, absolute agreement, single measure, calculated with the “irr” R package; Koo & Li, 2016). The ICC coefficient values were as follows: utterances on the literal level (.91), inferential emotional level (.94), inferential cognitive level (.97), and inferential non-mental level (.93).

### Data Analysis Strategy

The first and second hypotheses, regarding the group comparison between the child’s ToM abilities and content of parental talk (number of literal and inferential utterances—cognitive, emotional, and non-mental), were investigated using descriptive statistics and the nonparametric Wilcoxon rank-sum test. The choice of that test was determined by the fact that Wilcoxon rank-sum test does not assume that the samples are normally distributed (that was the case for some of the variables in our study) as well as it shows robustness for small and unequal sample sizes (Mann & Whitney, 1947). As the children with CIs were statistically older than children with TH (*p* < .001), only children with CIs who were less than 72 months old were selected for this stage of the analysis. The final subgroup for the first and second analyses consisted of 24 children with CIs (*M_age_* = 53.46, SD = 1.62) and 52 with TH (*M_age_* = 52.48, SD = 1.07).

The third hypothesis concerning the relation between ToM skills to parental MST were examined with non-parametric Spearman’s correlation analysis separately for each group. The analyses were carried out on the full sample of children with CIs and children with TH, described in the methods section.

The fourth hypothesis concerned the independent contribution of parental MST to the child’s ToM abilities when accounting for the child’s receptive grammar scores and age. To this end, we conducted hierarchical regression analyses with combined ToMI-2 score as a dependent variable. The analyses were carried out on the full sample of children with CIs, described in the methods section.

All analyses were carried out using R software (v4.1.0; R Core Team, 2021).

## Results

### Between-Group Comparisons

Group comparisons were performed on an age-matched sample of 24 children with CIs (*M_age_* = 53.46, SD = 1.62) and 52 with TH (*M_age_* = 52.48, SD = 1.07); however, since some of the children did not complete all tasks or parents did not complete the questionnaires, some comparisons were performed on smaller subgroups (see Table 3 note).

**Table 3:** Descriptive statistics and results of group comparisons with Wilcoxon Rank Sum Test

	CI group *(n = 24)*		TH group *(n = 52)*		
Variable	*M* (SD)	Range	*M* (SD)	Range	*r*
Age (in months)	53.5 (1.6)	35–67	52.5 (10.1)	36–70	.04
Receptive grammar	18.8 (6.5)	11–31	25.5 (5.7)	12–32	.45^***^
Verbal IQ	8.1 (1.9)	4–11	1.9 (2.5)	6–19	.52^***^
Non-verbal IQ	11.0 (2.0)	7–15	11.8 (2.5)	7–19	.09
Maternal education	16.2 (2.0)	12–18	18.7 (2.6)	14–26	.41^**^
Paternal education	16.3 (2.3)	11–19	18.2 (2.6)	13–26	.29^*^
Early ToM	16.2 (2.1)	12.6–19.3	17.5 (1.5)	13.1–19.5	.30^*^
Basic Tom	14.3 (2.3)	9.0–18.6	16.3 (1.8)	11.2–19.0	.40^***^
Advanced ToM	10.3 (3.1)	2.8–14.7	12.5 (2.5)	7.2–18.2	.32^**^
Combined ToM	13.2 (2.3)	8.3–17.3	15.1 (1.7)	1.6–18.8	.39^**^
Verbosity (no. words)	321.7 (207.9)	63–756	276.6 (21.4)	38–1,240	.12
Literal utterances	24.3 (18.6)	4–70	14.8 (15.9)	0–91	.30^*^
Emotional utterances	2.4 (3.6)	0–15	3.2 (3.1)	0–17	.18
Cognitive utterances	7.0 (6.6)	0–26	8.8 (7.5)	0–31	.13
Non-mental utterances	14.4 (11.0)	1–41	12.3 (9.8)	0–54	.09

^*^ *p* < .05.

^**^ *p* < .01.

^***^ *p* < .001.

Maternal and paternal education were measured in years of education; Basic, early, advanced and combined ToM relate to the scores from the Theory of Mind Inventory (ToMI-2; Hutchins & Prelock, 2016) and were performed on scaled scores; Maternal education data were not available for 5 children from the CI group and 11 children from the TH group; paternal education data were not available for 6 children from the CI group and 12 children from the TH group; ToMI-2 data were not available for 3 children from the TH group; IQ data were not available for 2 children from CI group and 5 children from TH group.

#### Receptive grammar, Child’s IQ, parental education

Group comparisons of background variables (receptive grammar, IQ, parents’ education) indicated that children with CIs obtained significantly lower scores for receptive grammar (*p* < .001) and verbal brief IQ (*p* < .001). There were no significant differences in nonverbal brief IQ (*p* = .453). There were significant differences for parental (maternal and paternal) education—parents of children with CIs reported fewer years of completed education than parents of children with TH (mothers: *p* = .002, fathers: *p* = .026).

#### Theory of mind (ToMI-2)

The analysis revealed significant differences for all ToMI-2 scales: early ToM (*p* = .011), basic ToM (*p* < .001), advanced ToM (*p* = .006) and the combined ToM scale (*p* = .001)—parents of children with CIs reported lower ToM abilities for their children than did parents of children with TH.

#### Shared storybook reading

Parents from the CI (*M_time_* = 9.33, SD = 3.42) and TH (*M_time_* = 8.29, SD = 2.69) groups did not differ in the time spent reading and talking about the book (*p* = .167). Parents did not differ in their verbosity, treated as the number of extratextual words spoken during shared storybook reading (*p* = .314). There were no significant differences between parents of children with CIs and children with TH in the number of utterances referring to cognitive (*p* = .274) or emotional mental states (*p* = .116) nor in inferential non-mental utterances (*p* = .447). However, there was a significant difference in the number of literal utterances (*p* = .01)—parents of children with CIs used more utterances of this type in comparison with parents of children with TH.

The summary statistics from abovementioned comparisons are presented in Table 3.

### Correlation Analyses

The analyses were carried out on the full sample of 39 children with CIs and 52 children with TH, described in the methods section.

#### CI group

Pairwise deletion method was used to deal with missing values in correlation analyses, since 1 child did not complete a receptive grammar test, three children did not complete the IQ assessment and one parent did not report on the child’s ToM.

In the CI group, parental emotional utterances were positively and moderately related to basic (*r_s_* = .40, *p* = .011), early ToM skills (*r_s_* = .33, *p* = .041) and combined ToM (*r_s_* = .37, *p* = .02). Parental cognitive utterances had a moderate positive relationship only with early ToM (*r_s_* = .32, *p* = .048). Finally, there was also a negative correlation between parental literal utterances and basic ToM (*r_s_* = −.36, *p* = .026). Grammar comprehension showed a moderate to high positive correlation with all ToM skills (.43 ≤ *r_s_* ≤ .72, *p* ≤ .01). It was also negatively correlated with literal utterances (*r_s_* = −.48, *p* = .002).

The results from the correlation analysis are reported in Table 4.

**Table 4:** Results of Spearman’s correlations for CI group

	Age	Receptive grammar	Nonverbal IQ	Early ToM	Basic ToM	Advanced ToM	Combined ToM	Literal utterances	Emotional utterances	Cognitive utterances
Age (in months)										
Receptive grammar	0.60^***^									
Nonverbal IQ	−0.22	0.10								
Early ToM	0.24	0.43^**^	0.05							
Basic ToM	0.54^***^	0.72^***^	−0.03	0.68^***^						
Advanced ToM	0.62^***^	0.67^***^	−0.04	0.65^***^	0.89^***^					
Combined ToM	0.57^***^	0.70^***^	0.01	0.73^***^	0.96^***^	0.97^***^				
Literal utterances	−.52^***^	−.48^**^	−0.09	−0.10	−0.36^*^	−0.29	−0.30			
Emotional utterances	−0.01	0.16	0.19	0.33^*^	0.40^*^	0.32	0.37^*^	0.28		
Cognitive utterances	−0.10	0.16	0.25	0.32^*^	0.28	0.24	0.27	0.27	0.62^***^	
Non-mental utterances	−0.39^*^	−0.22	0.13	0.06	−0.00	−0.13	−.07	0.62^***^	0.59^***^	0.72^***^

^*^ *p* < .05.

^**^ *p* < .01.

^***^ *p* < .001.

Basic, early, advanced, and combined ToM relate to the scores from the Theory of Mind Inventory (ToMI-2; Hutchins & Prelock, 2016) and were performed on scaled scores; receptive grammar data were not available for one child; IQ data were not available for 3 children; ToMI-2 data were not available for one child.

#### TH group

Similar to the analyses in the CI group, pairwise deletion method was used to deal with missing values, since four children did not complete the IQ assessment and three parents did not report on the child’s ToM.

There was no significant relationship between any ToM measures and MST in the TH group. However, similarly to the CI group, receptive grammar correlated significantly with basic, advanced and combined ToM skills (.37 ≤ *r_s_* ≤ .48, *p* ≤ .01). The results from the correlation analysis are reported in Table 5.

**Table 5:** Results of Spearman’s correlations for TH group

	Age	Receptive grammar	Nonverbal IQ	Early ToM	Basic ToM	Advanced ToM	Combined ToM	Literal utterances	Emotional utterances	Cognitive utterances
Age (in months)										
Receptive grammar	0.75^***^									
Nonverbal IQ	0.22	0.38^**^								
Early ToM	0.13	0.23	0.06							
Basic ToM	0.37^**^	0.48^***^	0.26	0.57^***^						
Advanced ToM	0.40^**^	0.37^**^	0.14	0.58^***^	0.76^***^					
Combined ToM	0.37^**^	0.40^**^	0.17	0.71^***^	0.90^***^	0.95^***^				
Literal utterances	−0.26	−0.20	−0.32^*^	−0.05	−0.17	−0.24	−.21			
Emotional utterances	−0.20	−0.17	0.08	0.13	−0.11	−0.24	−0.19	0.27		
Cognitive utterances	−0.06	0.03	0.07	0.20	0.09	−0.04	0.02	0.37^**^	0.64^***^	
Non-mental utterances	−0.14	−0.04	−0.24	0.00	−0.11	−0.14	−.13	0.61^***^	0.48^***^	0.63^***^

^*^ *p* < .05.

^**^ *p* < .01.

^***^ *p* < .001.

Basic, early, advanced, and combined ToM relate to the scores from the Theory of Mind Inventory (ToMI-2; Hutchins & Prelock, 2016) and were performed on scaled scores; IQ data were not available for 4 children; ToMI-2 data were not available for 3 children.

### Regression Analysis

As correlation analysis yielded positive relationships between ToM and the child’s age, receptive grammar, and parental MST referring to emotions (CI group only), these relationships were further examined. To this end, we performed hierarchical regression analyses with the combined ToMI-2 score (sum of basic, early, and advanced subscales) as the dependent variable, while receptive grammar score, child’s age, and parental emotional utterances were used as independent variables. The analyses were carried out only in the CI group (*n* = 39). However, two observations were excluded due to missing data on grammar comprehension and ToMI-2. Multicollinearity risk was assessed with the variance inflation factor (VIF)—all values were below 1.70 (value for child’s age) indicating low risk (James, Witten, Hastie, & Tibshirani, 2021).

In the first step, receptive grammar and the child’s age were entered as predictors of combined ToMI-2 score. The regression model was significant, Adj*. R*^2^ = .49, *F*(2,34) = 18.17, *p* < .001, with two predictors collectively explaining 49% of variance in ToM score. Receptive grammar (*β* = .48, *p* = .004) and child’s age (*β* = .31, *p* = .048) were both positive predictors of ToM scores. In the second step, parental emotional utterances were entered as a predictor. The regression model remained significant, Adj*. R*^2^ = .58, *F*(3,33) = 17.7, *p* < .001, with the three predictors jointly explaining 58% of the variance in ToM score. Receptive grammar (*β* = .41, *p* = .007), the child’s age (*β* = .35, *p* = .018), and parental emotional utterances (*β* = .32, *p* = .006) were significant predictors of ToM score. The second model with parental emotional utterances was significantly better at predicting ToM score than the first model, *F*(34,33) = 8.63, *p* = .006. The results from the models are reported in Table 6.

**Table 6:** Results of multiple hierarchical regression predicting combined ToM scores in children with CIs

			95% CI				
		*β*	LL	UL	*R^2^*	*Adjusted R^2^*	*F*
Step 1							
	Age (in months)	.31^*^	.00	.62			
	Receptive grammar	.48^**^	.17	.79			
					.52	.49	18.17^***^
Step 2							
	Age (in months)	.35^*^	.06	.63			
	Receptive grammar	.41^**^	.12	.69			
	Emotional utterances	.32^**^	.10	.54			
					.62	.58	17.70^***^

^*^ *p* < .05.

^**^ *p* < .01.

^***^ *p* < .001.

Receptive grammar data were not available for one child. ToMI-2 data were not available for one child.

## Discussion

Language may contribute to a child’s ToM development through the experience gained in everyday conversations with their parents, especially when parents discuss and explain mental states (Carpendale & Lewis, 2004; Devine & Hughes, 2018; Nelson et al., 2003).

Given the findings of previous studies, we were interested in exploring the relationship between the features of parental talk and ToM in children who may be at risk of early language deprivation due to deafness but who experience rapid hearing habilitation via cochlear implantation. Therefore, in the current study, we investigated parental MST, receptive grammar, ToM, and the relationships between them in deaf children who received a CI before the age of 21 months in comparison with their peers with typical hearing.

Overall, our results indicate qualitative differences between the conversational styles of parents of deaf children and children with typical hearing. Interestingly, they were not apparent in case of inferential utterances (mentalistic or non-mentalistic), but they did manifest for literal utterances. In addition, it was demonstrated that deaf children with CIs had lower ToM skills than hearing peers. Finally, the results yielded that parents of deaf children who talked more about the emotional states of others had children who displayed better ToM skills. In contrast, the propensity to use literal utterances was associated with poorer ToM capacities.

### ToM, Sentence Comprehension, and the Content of Parents' Mental State Talk

Consistent with previous studies (Pluta et al., 2021; Yu et al., 2021), our results provide support for the hypothesis that deaf children born to hearing parents experience ToM delays even if they received auditory assistance in the form of CIs early on in life (≤20 months of age). In this study, unlike other researchers, we did not use laboratory methods to measure ToM (which typically use false belief tasks, and thus only measure a selected aspect of this complex capacity), but instead we chose to use a caregiver-informant measure. As the ToMI-2 has been found to have good criterion-related validity (Hutchins & Prelock, 2016), we expected that parents provide reliable information on their children’s applied ToM competence in real-world context. Moreover, using this measure allowed us to assess the progression of various ToM-related skills that children manifest at different ages. Analysis of the results showed that deaf children scored significantly lower on all three subscales (early ToM, basic ToM, and advanced ToM) than their peers with TH. These findings are consistent with previous laboratory studies and imply that early auditory and hence language deprivation adversely affects many aspects of social functioning. First, it may impede the acquisition of affect recognition skills (measured here by the early ToM subscale) because this ability develops within the linguistic context (Lindquist, MacCormack, & Shablack, 2015). During typical development, children observe the facial expressions of others and also listen to others labeling and interpreting them (Wiefferink, Rieffe, Ketelaar, De Raeve, & Frijns, 2013). Deaf children born to hearing parents lack this verbal information before their hearing habilitation and access to language (Rieffe & Terwogt, 2006). Lower scores obtained by children with CIs on the basic and advanced subscales might be interpreted in the same vein. These subscales measure abilities that are known to be more dependent on the child's language skills (false belief understanding, second-order understanding of beliefs, recursive thinking, and metapragmatic understanding). Furthermore, deaf children with CIs showed reduced complex sentence comprehension (measured by TRJ), which might further hinder their acquisition of complex concepts related to ToM (in addition to an adverse language environment early in life).

In terms of MST, contrary to our hypotheses, the results showed no group differences in the number of utterances referring either to emotions or cognition. This suggests that children in the CI group had similar opportunities to learn about mental states during communicative exchanges with their parents while shared book reading as did their peers with TH. These results differ from the previous research, which reported significantly less MST provided by hearing parents of deaf children with CIs compared to parents of children with typical hearing (Moeller & Schick, 2006; Morgan et al., 2014). However, this result may be explained by substantial differences between previous studies and the current one in terms of group characteristics. In the study by Moeller and Schick (2006), parents communicated in sign language and the age of implantation of the children ranged from 36 to 96 months of age. In our study, all children received their first CI ≤ 20 months of age, which is considerably earlier. Thus, earlier implantation is one of the factors that might have positively contributed to children’s access to conversations about mental states. In the study by Morgan et al. (2014), children received a CI at a similar age (12 to 29 months) as participants in our study and the authors still reported fewer references to cognitions in the mothers’ narratives. These differences may be due to the age of the participants and parents’ fluency in bimodal communication. First, the children were younger than in our study. Secondly, although parents used both sign and spoken language to communicate with their children, they were not native speakers of sign language and their sign language experience varied significantly (range 2–15 years). Given that parental MST is related to parents’ language proficiency (Moeller & Schick, 2006) caregivers' sign proficiency may have influenced the results. In our study, parents and children were communicating only in spoken language; therefore, parents could talk about mental states without being constrained by their language abilities. Furthermore, the ecological validity of the shared storybook reading should be considered when interpreting the results. Although Tompkins et al. (2018) did not find significant differences between naturalistic observations and storybook reading, the parents in our sample might have tried to infer the aim of the study from its description or the measures used. Consequently, they may have talked more about mental states in the laboratory setting, irrespective of their child’s ToM and conversational abilities.

Conversely, our results show that parents of children with CIs provided more literal utterances directly referring to the observable contents of the book than did parents of children with TH. It may be the case that parents of deaf children with CIs were using literal references to match the perceived level of their child’s ToM abilities in order to support their development (Jakubowska et al., 2018; Morgan et al., 2014). Alternatively, it might also simply reflect the strategies they were instructed to apply by clinicians—bathing their children in language and narrating everything they see.

### Parents' Mental State Talk and Children's Theory of Mind

The analysis yielded that parents' MST was significantly related to children’s ToM, including both early and later developing skills, but only in dyads of deaf children with CIs and their hearing parents. These results highlight the facilitating effect of access to parental abstract mind-related discourse for the child’s social development, particularly for deaf children with CIs. Our results are in line with previous studies suggesting that in order to be optimally stimulating for ToM, conversations need to include concepts that refer to non-observable states of mind (Devine & Hughes, 2019; Tompkins et al., 2018). One plausible mechanism for this relationship is as follows: words referring to mental states such as “think” provide children labels for concepts that cannot be seen (Howard et al., 2008) and draw attention to the fact that mental states are subjective. Another mechanism is the embedding of cognitive terms in complement sentences (e.g., “Sally thinks that the marble is in the basket”), which emphasizes the possible contrast between beliefs and reality (de Villiers & de Villiers, 2000). This may be particularly critical for children with CIs (even with early implantation onset), who are at risk of delayed ToM development as the current and previous studies have shown (Pluta et al., 2021). We found positive associations between utterances referring to emotions and basic and early ToM subscales. Utterances referring to cognitions were correlated only with the early ToM subscale. This is somewhat inconsistent with the metaanalysis of Tompkins et al. (2018), which showed that cognitive state talk was a stronger predictor of false belief and emotional understanding than talk about desires and emotions. However, meta-analysis included only children with typical hearing levels and without developmental disabilities. In addition, both false belief and emotional understanding were tested in laboratory settings with the use of instruments that examine only selected ToM-related abilities. Therefore, the results cannot be directly compared.

Interestingly, our results indicated a negative correlation between the number of utterances relating to literal descriptions of an object, scene, or character and outcome on the basic ToM subscale. This may support the idea that the more often parents draw the child's attention to observable phenomena (perceptual properties of objects; e.g., color, size) the less the children focus their attention on unobservable and subjective mental states of others. Therefore, they may be less inclined to take other people's beliefs (including the false ones) into account when predicting their behavior. Given that the basic ToM subscale reflects how parents assess the development of their children's ability to understand the mental states of others, such a relationship seems possible.

An unexpected result of our analyses, in contrast to some other work, (Devine & Hughes, 2019; Tompkins et al., 2018), is the lack of an association between MST and ToM development in the TH group. Nevertheless, although previous studies indicated an association between MST and ToM, this effect was weak according to the meta-analysis (Devine & Hughes, 2016). The highest effect sizes for MST and ToM relationship were observed in short longitudinal studies (i.e., > 1 year) (Tompkins et al., 2018). According to the authors, this reflects the time necessary for children to internalize the capacities that are scaffolded in communicative exchanges.

However, a result similar to ours was obtained by Moeller and Schick (2006), who also studied hearing preschoolers (ages 4–6). They concluded that the lack of an association between MST and ToM may be due to the high performance of children with typical hearing and the age range. Given the smaller range of ToM scores in the TH group than the CI group and the restricted age of children who took part in the present study, these results can be interpreted in a similar way. Thus, it may be argued that the facilitatory role of MST on ToM development is more prominent in children who are at risk of ToM dysfunctions or delays. In such cases, richly mind-focused conversion might benefit children’s ToM growth.

### Predictors of ToM Development in Deaf Children with CIs

Our findings from regression analysis supported the hypothesis that receptive grammar and age predicted ToM development in children with CIs. This is in agreement with previous studies that concluded that ToM depends on language and particular competencies associated with ToM are age dependent (Wellman, Cross, & Watson, 2001; Wellman, Fang, & Peterson, 2011). Interestingly, the analysis indicated that MST referring to emotions explained additional variance in ToM. This suggests that although DoH children have reduced early access to language, which can contribute to early receptive language deficits delaying theory of mind development, enhanced exposure to MST may assist in developmental progressions of ToM in deaf children with CIs.

Of particular importance may be utterances referring to emotions. This may be related to the fact that emotional states (e.g., joy, sadness) rather than cognitive mental states (thoughts) are accompanied by observable expressions (laughing, crying), so it may therefore be easier for the caregiver to draw the child's attention to other people's internal mental states (e.g., “Look, Lucy is sad and her tears are falling”).

## Conclusions

This study investigated access to mental state talk in preschool-aged deaf children who use cochlear implants and is one of the first studies to examine talk about mental states in this cohort. Using a caregiver-informant measure of diverse ToM abilities, we showed that deaf children (even with cochlear implantation ≤20 months of age) born to hearing parents underperform in comparison with their hearing peers during every day social interactions that require understanding of mental states of others. This might be accounted for (at least partially) by poorer receptive grammar, as indicated by the results of the Sentence Comprehension Task. The present study also revealed qualitative differences in the conversational style of parents of children with CIs and typical hearing. Although parents in both groups employed comparable numbers of utterances referring to emotions and cognitive states, parents of deaf children were more likely to refer to observable phenomena then parents of children with typical hearing during shared book reading. Because of the negative relationship between ToM abilities and utterances referring to perception revealed in the correlation analysis, it can be proposed that they are thus directing the child's attention more to observable phenomena (e.g., properties of objects). This strategy may be less favorable for acquiring an understanding that other people may have different mental states.

The study also highlighted the importance of parental input (especially utterances referring to emotions) for socio-cognitive development of deaf children with CIs.

The results of the current study also have practical applications—our findings can be used in the development of therapeutic programs for clinicians, teachers and parents of deaf children, which emphasize the role of MST in supporting the development of theory of mind. This is especially important for this particular group of children, who are at risk of delayed ToM development due to early language deprivation.

## Limitations

This study has some limitations. Since children from the CI group were significantly older than their peers with TH, group comparisons were performed for a smaller subgroup of participants matched in age to the TH group (i.e., age 3–5 years). Moreover, MST was only measured during shared storybook reading. Although the chosen book provided several opportunities to talk about mental states, it might be important to investigate MST in deaf children with CIs across various contexts, also in home settings, including the use of non-structured play. Another drawback of the study is that parents of children with CI and TH differed in their educational background—parents of children with TH had, on average, higher levels of education. This may result from the fact that parents of deaf children could have been more motivated to have their children participate in a scientific study that measured language and cognitive abilities. Whereas, perhaps in the case of parents of children with TH, those whose motivation was to solely assist scientific research were most likely to volunteer for the study. Nevertheless, it should be noted that in the CI group, mothers had at least 12 years of education and over 70% had more than 15 years of education meaning that it is rather unlikely that differences in education between groups could have compromised parental understanding of the measurements used in the study.

## Funding

This work was supported by the National Science Centre [2017/25/B/HS6/01624] granted to A.P. and by the Faculty of Psychology, University of Warsaw, from the funds awarded by the Ministry of Science and Higher Education in the form of a subsidy for the maintenance and development of research potential in 2022 (501-D125-01-1250000 zlec 5011000251). 

## Conflict of Interest

None declared

## Data Availability

The data underlying this article will be sharedon reasonable request to the corresponding author.
